# Supplementary material for: Gut Microbiota in Chronic Kidney Disease: From Composition to Modulation towards Better Outcomes—A Systematic Review
Source: J Clin Med. 2023 Mar 1;12(5):1948. doi: 10.3390/jcm12051948 (PMC10003930; doi:10.3390/jcm12051948)
Supplement: Supplementary file 1 [file jcm-12-01948-s001.zip › Table S1. Databases and search strategy.pdf]

Table S1. Databases and search strategies used in present systematic review.

Database	Coverage	Search run	Records
**MEDLINE**	1946 to present	"(microbiota OR microbiome OR microflora) AND (gut OR intestinal OR gastrointestinal) AND (composition OR diversity) AND (chronic kidney disease OR end-stage kidney disease OR haemodialysis)"	311
		"(microbiota OR microbiome OR microflora) AND (gut OR intestinal OR gastrointestinal) AND (composition OR diversity) AND (chronic kidney disease OR end-stage kidney disease OR haemodialysis) AND (renal decline OR disease severity OR progression OR mortality OR major adverse cardiovascular events OR inflammation OR inflammatory response) AND (probiotic OR prebiotic OR symbiotic)"	52
		"("Gastrointestinal Microbiome"[Mesh]) AND "Renal Insufficiency, Chronic"[Mesh]"	312
			Total records = 675
**Embase**	1966 to present	"(microbiota OR microbiome OR microflora) AND (gut OR intestinal OR gastrointestinal) AND (composition OR diversity) AND (chronic kidney disease OR end-stage kidney disease OR haemodialysis)"	511
		"(microbiota OR microbiome OR microflora) AND (gut OR intestinal OR gastrointestinal) AND (composition OR diversity) AND (chronic kidney disease OR end-stage kidney disease OR hemodialysis) AND (renal decline OR disease severity OR progression OR mortality OR major adverse cardiovascular events OR inflammation OR inflammatory response) AND (probiotic OR prebiotic OR symbiotic)"	16
		"(microbiota OR microbiome OR microflora) AND (chronic kidney disease OR end-stage kidney disease OR haemodialysis)"	199
			Total records = 726
**Cochrane library**	1967 to present	"(microbiota OR microbiome OR microflora) AND (gut OR intestinal OR gastrointestinal) AND (composition OR diversity) AND (chronic kidney disease OR end-stage kidney disease OR haemodialysis)"	52
		"(microbiota OR microbiome OR microflora) AND (gut OR intestinal OR gastrointestinal) AND (composition OR diversity) AND (chronic kidney disease OR end-stage kidney disease OR hemodialysis) AND (renal decline OR disease severity OR progression OR mortality OR major adverse cardiovascular events OR inflammation OR inflammatory response) AND (probiotic OR prebiotic OR symbiotic)"	16
		"(microbiota OR microbiome OR microflora) AND (chronic kidney disease OR end-stage kidney disease OR haemodialysis)"	187
			Total records = 255
**Scopus**	From the inception till present	"(microbiota OR microbiome OR microflora) AND (gut OR intestinal OR gastrointestinal) AND (composition OR diversity) AND (chronic kidney disease OR end-stage kidney disease OR haemodialysis)"	348

	"(microbiota OR microbiome OR microflora) AND (gut OR intestinal OR gastrointestinal) AND (composition OR diversity) AND (chronic kidney disease OR end-stage kidney disease OR hemodialysis) AND (renal decline OR disease severity OR progression OR mortality OR major adverse cardiovascular events OR inflammation OR inflammatory response) AND (probiotic OR prebiotic OR symbiotic)"	0
	"(microbiota OR microbiome OR microflora) AND (chronic kidney disease OR end-stage kidney disease OR haemodialysis)"	1281
		Total records = 1629
All databases: 3285 records		
