# Supplementary material for: Gut Microbiota in Chronic Kidney Disease: From Composition to Modulation towards Better Outcomes—A Systematic Review
Source: J Clin Med. 2023 Mar 1;12(5):1948. doi: 10.3390/jcm12051948 (PMC10003930; doi:10.3390/jcm12051948)
Supplement: Supplementary file 1 [file jcm-12-01948-s001.zip › Tables S2-S4. Quality assessment..pdf]

Table S2. Quality assessment of cross-sectional using Newcastle-Ottawa scale.

Study	Representative-ness of the sample	Sample size	Non-response rate	Ascertainment of the exposure (risk factor)	Comparability	Assessment of the outcome	Statistical test	Total
Barros, 2015	*	*	NA	*	**	*	*	7
Chen, 2021	*	*	NA	*	**	*	*	7
Du, 2021	*	*	NA	*	**	*	*	7
Gao, 2021	*	*	NA	*	*	*	*	6
Gryp, 2021	*	*	NA	*	*	*	*	6
Gryp, 2020	*	*	NA	*	*	*	*	6
Guirong, 2018	*	*	NA	*	*	*	*	6
Hanifi, 2021	*	*	NA	*	*	*	*	6
He, 2020	*	*	NA	*	*	*	*	6
He, 2021	*	*	NA	*	**	*	*	7
Hu et, 2020	*	*	NA	*	**	*	*	7
Hu, 2022	*	*	NA	*	**	*	*	7
Jiang, 2017	*	*	NA	*	**	*	*	7
Khiabani, 2022	*	*	NA	*	*	*	*	6
Kim, 2020	*	*	NA	*	**	*	*	7
Kumar, 2021	*	*	NA	*	**	*	*	7
Lun, 2018	*	*	NA	*	*	*	*	6
Margiotta, 2020	*	*	NA	*	**	*	*	7
Al-Obaide, 2017	*	*	NA	*	*	*	*	7
Salguero, 2019	*	*	NA	*	**	*	*	7
Sato, 2021	*	*	NA	*	**	*	*	7
Stadlbauer, 2017	*	*	NA	*	**	*	*	7
Wang, 2019	*	*	NA	*	**	*	*	7
Wang et al., 2020	*	*	NA	*	*	*	*	6
Wu, 2020	*	*	NA	*	**	*	*	7
Wu, 2020	*	*	NA	*	**	*	*	7
Wu, 2020	*	*	NA	*	**	*	*	7
Wu, 2021	*	*	NA	*	**	*	*	7
Wu, 2020	*	*	NA	*	**	*	*	7
Xu, 2017	*	*	NA	*	*	*	*	6
Zhang, 2021	*	*	NA	*	*	*	*	6
Zhang, 2020	*	*	NA	*	**	*	*	7
Zhou, 2022	*	*	NA	*	*	*	*	6
Zhu, 2022	*	*	NA	*	**	*	*	7

NA = not applicable.

Good quality: 3 or 4 stars in selection domain AND 1 or 2 stars in comparability domain AND 2 or 3 stars in outcome/exposure domain. Fair quality: 2 stars in selection domain AND 1 or 2 stars in comparability domain AND 2 or 3 stars in outcome/exposure domain. Poor quality: 0 or 1 star in selection domain OR 0 stars in comparability domain OR 0 or 1 stars in outcome/exposure domain.

Table S3. Quality assessment of case-control studies using Newcastle-Ottawa scale.

Study	Case definition	Case representativeness	Selection of controls	Definition of controls	Comparability	Exposure ascertainment	Ascertainment methods	Non-response rate	Total
Hu, 2020	*	*	*	*	*	*	*		7
Hu, 2020	*	*	*	*	*	*	*		7
Jiang, 2016	*	*	*	*	*		*		6
Lin, 2020	*	*	*	*	*	*	*		7
Lin, 2020	*	*	*	*	*	*	*		7
Lin, 2022	*	*	*	*	*	*	*		7
Liu, 2021	*	*	*	*	*	*	*		7

Good quality: 3 or 4 stars in selection domain AND 1 or 2 stars in comparability domain AND 2 or 3 stars in outcome/exposure domain. Fair quality: 2 stars in selection domain AND 1 or 2 stars in comparability domain AND 2 or 3 stars in outcome/exposure domain. Poor quality: 0 or 1 star in selection domain OR 0 stars in comparability domain OR 0 or 1 stars in outcome/exposure domain.

Table S4. Quality assessment of cohort studies using Newcastle-Ottawa scale.

Study	Representativeness of the exposed cohort	Selection of the non-exposed cohort	Ascertainment of exposure	Presence of outcome of interest at the start	Comparability of cohorts	Assessment of outcome	Follow-up long enough for outcomes to occur	Adequacy of follow-up	Total
Abdelbary, 2022	*	*	*	*		*	*		6
Iguchi, 2020	*	*	*	*	*	*	*		7
Jiang, 2020	*	*	*	*		*	*		6
Lai, 2019	*	*	*	*	*	*	*		7
Lecam-wasam, 2021	*	*	*	*	**	*	*		8
Li, 2019	*	*	*	*	**	*	*		8
Lin, 2021	*	*	*	*	*	*	*		7
Liu, 2022	*	*	*	*	*	*	*		7
Luo, 2021	*	*	*	*	**	*	*		8
Miao, 2018	*	*	*	*		*	*		6
Nazzal, 2017	*	*	*	*	*	*	*		7
Pivari, 2022	*	*	*	*	**	*	*		8
Ren, 2020	*	*	*	*	*	*	*		7
Wang, 2019	*	*	*	*	*	*	*		7
Yacoub, 2017	*	*	*	*	*	*	*		7
Zheng, 2020	*	*	*	*	*	*	*		7

Good quality: 3 or 4 stars in selection domain AND 1 or 2 stars in comparability domain AND 2 or 3 stars in outcome/exposure domain. Fair quality: 2 stars in selection domain AND 1 or 2 stars in comparability domain AND 2 or 3 stars in outcome/exposure domain. Poor quality: 0 or 1 star in selection domain OR 0 stars in comparability domain OR 0 or 1 stars in outcome/exposure domain.
